# Catalytic deficiency of O-GlcNAc transferase leads to X-linked intellectual disability

**DOI:** 10.1073/pnas.1900065116

**Published:** 2019-07-11

**Authors:** Veronica M. Pravata, Villo Muha, Mehmet Gundogdu, Andrew T. Ferenbach, Poonam S. Kakade, Vasudha Vandadi, Ariane C. Wilmes, Vladimir S. Borodkin, Shelagh Joss, Marios P. Stavridis, Daan M. F. van Aalten

**Affiliations:** ^a^Division of Gene Regulation and Expression, School of Life Sciences, University of Dundee, DD1 5EH Dundee, United Kingdom;; ^b^Division of Cell and Developmental Biology, School of Life Sciences, University of Dundee, DD1 5EH Dundee, United Kingdom;; ^c^West of Scotland Genetic Service, Queen Elizabeth University Hospital, G51 4TF Glasgow, United Kingdom

**Keywords:** intellectual disability, O-GlcNAc, neurodevelopment

## Abstract

O-GlcNAc transferase (OGT) is an X-linked gene product that is essential for normal development of the vertebrate embryo. It catalyses the O-GlcNAc posttranslational modification of nucleocytoplasmic proteins and proteolytic maturation of the transcriptional coregulator Host cell factor 1 (HCF1). Recent studies have suggested that conservative missense mutations distal to the OGT catalytic domain lead to X-linked intellectual disability in boys, but it is not clear if this is through changes in the O-GlcNAc proteome, loss of protein–protein interactions, or misprocessing of HCF1. Here, we report an OGT catalytic domain missense mutation in monozygotic female twins (c. X:70779215 T > A, p. N567K) with intellectual disability that allows dissection of these effects. The patients show limited IQ with developmental delay and skewed X-inactivation. Molecular analyses revealed decreased OGT stability and disruption of the substrate binding site, resulting in loss of catalytic activity. Editing this mutation into the *Drosophila* genome results in global changes in the O-GlcNAc proteome, while in mouse embryonic stem cells it leads to loss of O-GlcNAcase and delayed differentiation down the neuronal lineage. These data imply that catalytic deficiency of OGT could contribute to X-linked intellectual disability.

## Significance

Protein O-GlcNAcylation is a posttranslational modification essential for development. Recently, mutations in the O-GlcNAc transferase (OGT) substrate binding domain have been described that lead to intellectual disability, but the mechanisms underpinning pathogenesis remain to be explored. This work describes the first point mutation in the OGT catalytic domain leading to effects on O-GlcNAcylation and Host cell factor 1 processing in vitro and in a stem cell/*Drosophila* model, resulting in delayed neuronal differentiation. This establishes a potential link between OGT activity and intellectual disability.

The O-linked β-*N*-acetyl-d-glucosamine (O-GlcNAc) transferase (OGT) and the hydrolase O-GlcNAcase (OGA) together orchestrate protein O-GlcNAcylation, a dynamic co/posttranslational modification cycling on thousands of nucleocytoplasmic proteins (1, 2). OGT catalyses the covalent attachment of the monosaccharide O-GlcNAc to serine or threonine. This modification has been suggested to affect transcription (3–6), translation (1), protein stability (7, 8), and subcellular localization (9, 10). O-GlcNAcylation plays a key role in regulating stress response (11–13), differentiation (14, 15), nutrient sensing (16, 17), and autophagy (18).

Genetic studies have highlighted the importance of OGT and protein O-GlcNAcylation in development. OGT is essential for embryonic stem cell viability (19, 20) and mouse embryonic development (21). Zebrafish lacking OGT function exhibit shortened body axis and smaller brains (22). The *Drosophila melanogaster* OGT encoded by the Polycomb group gene *super sex combs* (*sxc*) plays a critical role in establishing *Drosophila* segment identity (23, 24).

The O-GlcNAc modification is particularly abundant in the brain (25, 26), where it controls memory formation (27–29), circadian rhythm (30, 31), and appetite (32, 33). Numerous neuron-specific proteins are O-GlcNAcylated, such as the Microtubule-associated proteins tau (34) and CRMP2 (35), synaptic vesicle proteins (36), the transcriptional factor cyclic-AMP response element binding protein (CREB) (28), and the AMPA receptor GluA2 subunit (27). Growing evidence suggests that O-GlcNAcylation is essential for normal development and function of the mammalian nervous system (21, 33, 37–39).

Although O-GlcNAcylation has been long implicated in chronic metabolic diseases, such as type II diabetes mellitus, neurodegeneration, and cancer, its role in neurodevelopmental disorders has only recently become apparent. In the past year, a small number of missense mutations within the human OGT gene have been discovered in patients with X-linked intellectual disability (XLID) (40–43). Intellectual disability (ID) refers to a broad range of developmental disorders characterized by limited intellectual capacity, IQ below 70, and poor adaptive behavior with onset before the age of 18, affecting 1 to 3% of the population worldwide (44). Genetic factors are the major cause of this developmental condition involving over 700 ID genes (45). These genes encode for proteins that are required for neuronal development and activity, contributing to neuronal structure and function through several signaling pathways (46). Mutations in genes located on the X-chromosome account for ∼5 to 10% of all ID causative genes affecting predominantly male individuals (44, 47).

To date, XLID-associated mutations in the OGT gene have only been reported for male patients, causing developmental delay and severe cognitive disability. Accompanying clinical phenotypes were dysmorphic features, such as clinodactyly, eye abnormalities, and microcephaly. A common molecular trait of the missense XLID OGT variants is that they appear to retain substantial OGT catalytic activity. This is in agreement with these mutations all being localized to the C-terminal noncatalytic tetratricopeptide repeat (TPR) domain of OGT that is responsible for substrate specificity and scaffolding (40–43). In cultured cells, global O-GlcNAc levels on proteins were unaltered (41–43), and a decrease in OGA protein expression to compensate for the (presumed) moderate loss of OGT activity was only apparent in patient-derived fibroblast cells (43). In addition to its glycosyltransferase activity, mammalian OGT is involved in the unusual proteolytic maturation of Host cell factor 1 (HCF1) (48–50), a transcriptional cofactor implicated in cell cycle control (51), also identified as an ID gene (52–54). HCF1 is O-GlcNAc–modified and subsequently cleaved by OGT using the same active site for both enzymatic activities (49). Among XLID variant OGT models, a moderate effect on HCF1 processing was only detected for one of the mutations (41, 43).

From the currently available OGT XLID mutations, it remains unclear if the patient phenotypes observed are linked to changes in the O-GlcNAc proteome, loss of protein–protein interactions, or misprocessing of HCF1. Here, we characterize an OGT missense mutation in the catalytic domain as observed in female twins with ID and developmental delay. In vitro biochemical analysis of recombinant N567K OGT missense variant protein revealed that this mutation abrogates OGT and HCF1 proteolytic processing activity. Protein crystallography data explain the molecular basis of the observed loss of substrate binding. Introduction of this mutation into *D. melanogaster* by genome editing reveals global effects on the O-GlcNAc proteome. In mouse embryonic stem cells (mESCs) carrying this mutation, loss of OGA protein expression was observed, as well as defective HCF1 processing. The mutation caused defects in neurite outgrowth during neuronal differentiation. Taken together, our data provide evidence that loss of OGT activity could contribute to the XLID phenotype observed in the patients.

## Results and Discussion

### Monozygotic Twins with ID and Developmental Delay Possess a De Novo OGT Mutation.

Twin girls (twin 1 and twin 2) were born at 33-wk gestation by semielective caesarean section for maternal preeclampsia. Conception was unassisted. Twin 1 weighed 1,670 g at birth and required continuous positive airway pressure (CPAP) to aid breathing for 3 wk and supplemental oxygen for 58 d. Twin 2 weighed 2,150 g and needed CPAP for 2 d and supplemental oxygen for 20 d. Both babies were tube-fed initially but bottle feeding was established from 3 to 4 wk of age. The twins showed delay in reaching developmental milestones, especially in areas of speech and language development. By 4 y of age, twin 1 only used 3 to 4 single words; at 10 y she was able to put 2 to 3 words together. Language development of twin 2 was more advanced than her sister; at 4 y she was using short phrases and at 10 y she was speaking in sentences and able to recognize letters of the alphabet. Their movement and physical development were also affected: twin 1 by age 4 y and twin 2 by the age of 2 y were able to walk independently. However, twin 2 had significant gross motor difficulties and clumsiness at the age of 8 y. Both twins were diagnosed with cerebral visual impairment, twin 1 more severe than twin 2. They have attended an additional needs nursery and school.

**Fig. 1.**
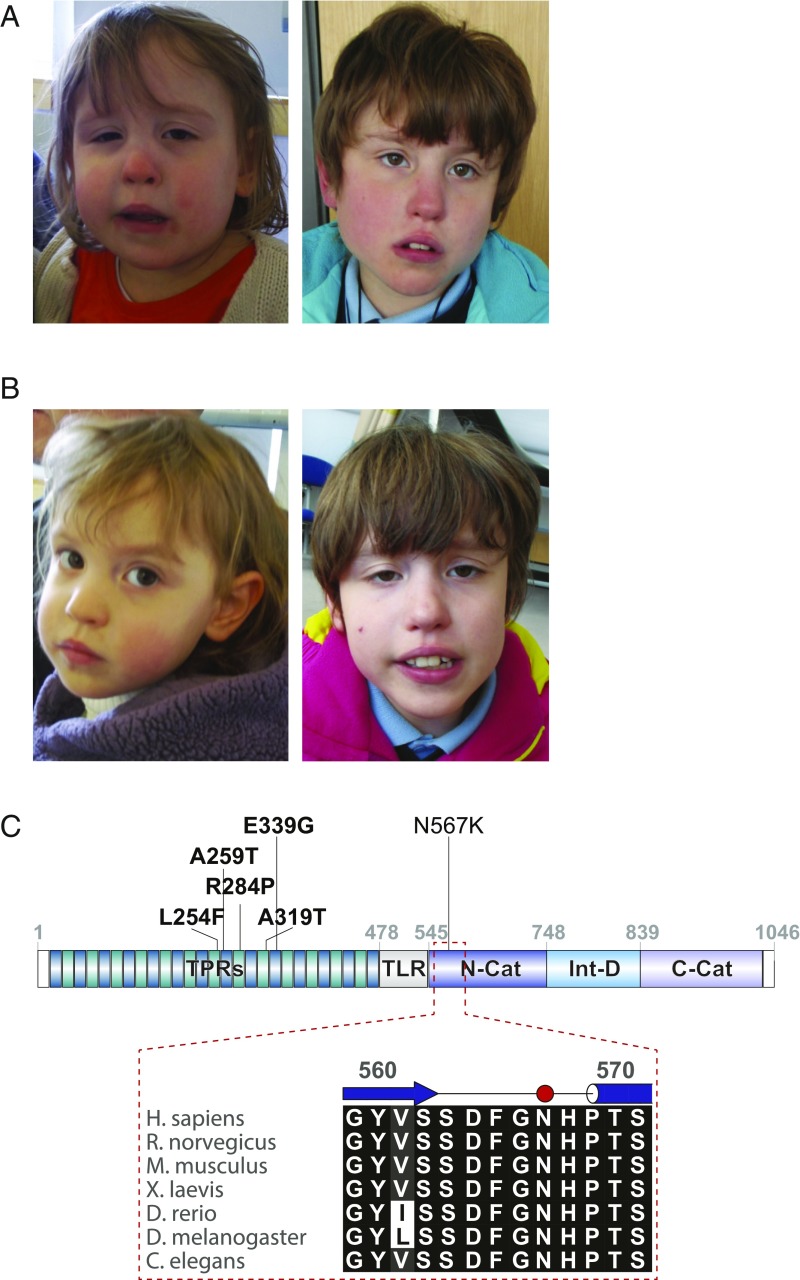
Clinical images from patients with the N567K mutation in the catalytic domain of OGT. (*A*) Picture of twin 1 showing dysmorphic features at age 4 y (*Left*) and 10 y (*Right*). (*B*) Picture of twin 2 showing dysmorphic features at age 4 y (*Left*) and 10 y (*Right*). (*C*) Schematic diagram of OGT highlighting the TPRs, TPR-like repeat, N-Cat, and C-Cat, and the site of N567K, as well as the previously identified XLID-associated mutations in OGT. The *Inset* shows the sequence alignment of the region encompassing the N567K mutation across the commonly used model organisms. N-Cat and C-Cat: N- and C-terminal lobes of OGT catalytic domain; TPR: tetratricopeptide repeat domain; TLR: tetratricopeptide repeat-like domain.

The twins (Fig. 1 *A* and *B*) appeared to be affected by a similar developmental syndrome, including small nose, high arched palate, and fifth finger clinodactyly. Twin 1 has ataxic gait with knees bent, walking on toes with inverted feet. An MRI brain scan showed prominence of the lateral ventricles and an enlarged cistern magna in twin 1 at age 2 y, suggesting possible hypoplasia of the inferior cerebellar vermis. Although both siblings have kept generally good health, hypotonia was apparent in infancy and they required iron supplementation intermittently. Twin 1 had an adenoidectomy and treatment for gastro-esophageal reflux and constipation.

Sanger sequencing was performed as part of the Deciphering Developmental Disorders initiative, which currently lists genomic and clinical data from over 14,000 child patients with severe undiagnosed developmental disorders (55). This revealed a single pathogenic missense mutation in the OGT gene (X:70779215 T > A, N567K). The mutation was found absent in control population of 141,456 samples, comprising 125,748 exome sequences and 15,708 whole-genome sequences from unrelated individuals reported in the genome aggregation database, *gnomAD* (56). The mutation affected both monozygotic twins, whereas both of their parents were noncarriers. A 98:2 skew in X-inactivation was detected in both children using polymorphic markers that are differentially methylated on the active and inactive chromosome (*SI Appendix*, Fig. S1). However, being a de novo mutation, which 1 of the 2 chromosomes is predominantly inactivated remains unclear. Taking these data together, we have identified monozygotic twins with ID and developmental delay that possess a de novo OGT mutation.

### The N567K Mutation Abrogates OGT Activity In Vitro.

**Fig. 2.**
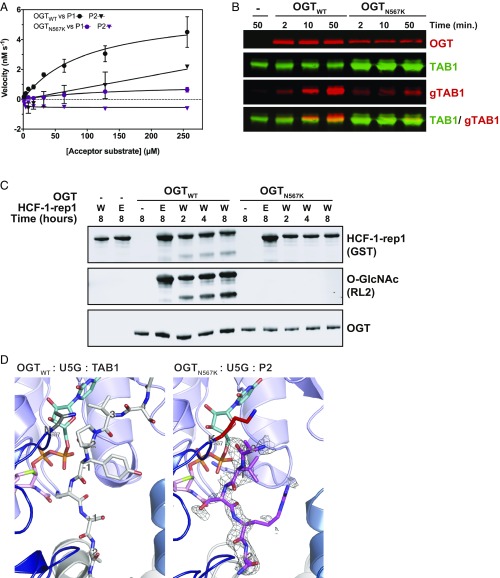
The N567K mutation abrogates OGT activity due to disrupted acceptor binding site. (*A*) Michaelis–Menten kinetics of OGT glycosyltransferase activity against P1 and P2 peptides. (*B*) Immunoblots showing OGT glycosyltransferase activity against TAB1. (*C*) Immunoblots showing OGT glycosyltransferase and proteolytic activities against HCF1-rep1. HCF1-rep1: GST-tagged host cell factor-1 fragment containing the first PRO repeat. W: wild type. E: E1019Q. (*D*) Crystal structures of OGT ternary complexes of OGT_WT_ and OGT_N567K_ active site in complex with UDP-5S-GlcNAc (U5G, turquoise, orange atoms) and acceptor peptide. OGT_WT_ is shown in complex with RB2 (Retinoblastoma-like protein 2 peptide, light gray carbon atoms) [PDB ID code: 5C1D (57)]; OGT_N567K_ is shown with P2 peptide (pink carbon atoms) (PDB ID code 6IBO). K567 is shown in red. An unbiased *F*o–*F*c difference map before inclusion of the peptide is shown as a mesh.

To understand the potential effects of the OGT N567K (OGT_N567K_) mutation, we next investigated changes in catalytic activity compared with full-length wild-type OGT (OGT_WT_) protein. Steady-state kinetics of OGT glycosyltransferase activity was measured against 2 acceptor peptide substrates, P1 (KKVPVSRA) and P2 (KKVAVSRA), that only varied at the −2 position adjacent to the O-GlcNAcylation site (57). OGT_N567K_ activity was 12-fold reduced against P1 (Fig. 2*A* and Table 1) and not detectable against P2 (Fig. 2*A*). Next, OGT activity was measured against an intact protein substrate, TAK-1 binding protein (TAB1), where the reaction was followed with a TAB1 O-GlcNAc site-specific antibody (58). OGT_N567K_ showed negligible glycosyltransferase activity (Fig. 2*B*).

**Table 1.** Michaelis–Menten kinetics of OGT activity against P1 peptide substrate

Enzyme	*K*_M_ (nM⋅s^−1^)	*k*_cat_ (s^−1^)	*k*_cat_/*K*_M_
OGT_WT_	99 ± 19	119 ± 11	1.2 ± 0.3
OGT_N567K_	144 ± 63	20 ± 4	0.1 ± 0.2

OGT possesses a second catalytic activity in the form of promoting autocatalytic cleavage and activation of HCF1, which itself is an ID-associated gene (52). We next investigated the effects of the N567K mutation on OGT-mediated HCF1 processing. OGT proteolytic activity was measured using a GST-fusion construct of a minimal HCF1 fragment (HCF1-rep1) containing one of the PRO repeats that are known to be the targets of OGT-mediated proteolysis (49). Wild-type OGT not only promotes HCF1 proteolysis but was also able to hyperglycosylate this protein fragment (Fig. 2*C*). HCF1_E1019Q_, which lacks the key glutamate required for proteolytic processing, was not cleaved by wild-type OGT_._ Strikingly, even after 8 h of reaction time, OGT_N567K_ was able to neither glycosylate nor cleave HCF1-rep1 (Fig. 2*C*). Taken together, the data show that the N567K mutation abrogates OGT activity.

### The N567K Mutation Disrupts the OGT Acceptor Binding Site.

To understand the molecular basis of the observed loss of OGT catalytic activity, we next initiated structural characterization of OGT_N567K_. Inspection of the wild-type OGT structure reveals that Asn567 maps to a loop in the N-terminal lobe of the catalytic domain that is completely conserved through evolution from *Caenorhabditis elegans* to man (Fig. 1*C*). The Asn567 side chain borders the −2 subsite as part of the OGT acceptor substrate binding cleft (Fig. 2*D*). Therefore, we hypothesized that the N567K mutation would affect OGT acceptor substrate binding, without affecting the overall OGT fold. Recombinant OGT_N567K_ was obtained from *Escherichia coli* using construct boundaries previously employed to crystallize OGT_WT_-substrate complexes (57). OGT_N567K_ was then cocrystallized with a donor analog, UDP-5S-GlcNAc, and 2 OGT acceptor peptide substrates, P1 (KKVPVSRA) and P2 (KKVAVSRA), previously used for activity measurements (Fig. 2*A*). Only the condition containing P2, which contains an alanine at the −2 subsite, yielded crystals that diffracted to 2.3 Å and allowed structure solution by molecular replacement and refinement (Table 2). The OGT_N567K_ structure superposed onto that of OGT_WT_ with an rmsd of 0.4 Å across 675 C_α_ atoms, suggesting the mutation does not induce large conformational changes in the catalytic domain. Initial unbiased difference electron density maps revealed partial density corresponding to the acceptor peptide (Fig. 2*D*). Surprisingly, this only covered the P2 backbone in the +2 through to −2 subsites, suggesting that the N567K mutation may induce increased flexibility at the N-terminal tail of the acceptor peptide (Fig. 2*D* and *SI Appendix*, Fig. S2). In agreement with this, a comparative B-factor analysis suggested that the acceptor peptide bound to OGT_N567K_ is substantially more flexible than that bound to OGT_WT_ (*SI Appendix*, Fig. S2). Close inspection of the OGT_N567K_ acceptor substrate binding cleft revealed that the protrusion caused by the N567K mutation partially occupies the position of the −2 proline frequently observed in OGT acceptor peptides (57, 59, 60). In summary, the N567K mutation appears to disrupt the OGT acceptor binding site.

**Table 2.** X-ray diffraction data processing and refinement statistics of the OGT_N567K_ ternary complex

	OGT_N567K_
Data collection	
Space group	*F*222
Cell dimensions	
α, β, γ (°)	137.7, 150.6, 200.0
A, B, C (Å)	90.0, 90.0, 90.0
Resolution (Å)	45.31–2.17 (2.17–2.23)
*R*_sym_ or *R*_merge_	0.07 (0.91)
*I*/σ*I*	11.3 (1.3)
Completeness (%)	100 (99)
Redundancy	5.2 (5.3)
Refinement	
Resolution (Å)	45.31–2.17 (2.17–2.23)
No. reflections	286,887 (23,302)
*R*_work_/*R*_free_	0.20/0.25
No. atoms	
Protein	5,530
Nucleotide sugar	39
Peptide	35
Water	328
*B*-factors	
Protein	53.2
Nucleotide sugar	46.6
Peptide	91.9
Water	51.3
Rmsd	
Bond lengths (Å)	0.02
Bond angles (°)	1.8

Numbers in brackets show the highest resolution bin.

### The *sxc*^*N595K*^ Mutation Leads to a Reduction of Protein O-GlcNAcylation in *D. melanogaster*.

Lack of functional OGT causes lethality and developmental arrest in most metazoan model organisms, including mice, frogs, zebrafish, and fruit flies (19, 22, 24, 61). To probe if the N567K mutation directly affects OGT catalytic activity in vivo, we exploited the genetically tractable system *D. melanogaster*, where phenotypes arisen due to null and hypomorphic alleles of *sxc* (the fly *ogt* ortholog) have been described (62, 63). *Drosophila sxc* null mutants (*sxc*^*1*^ and *sxc*^*6*^) die at the pharate adult stage with homeotic transformation defects (62). A hypomorph allele, *sxc*^*H537A*^, having substantially reduced OGT catalytic activity (64), shows significantly decreased protein O-GlcNAcylation levels (63) yet produce fertile offspring. Crucially, the fruit fly provides a platform to dissect the different effects of the OGT N567K mutation. In the fly, feedback between proteome O-GlcNAcylation levels and OGA/OGT protein/mRNA levels has not been observed. Furthermore, although *Drosophila* Hcf1 is heavily O-GlcNAc modified, proteolytic processing is catalyzed by the Taspase I protease instead of OGT (65).

We introduced the OGT N567K equivalent mutation (N595K) into *D. melanogaster* using a CRISPR/Cas9 gene-editing toolkit. Vasa::Cas9 embryos were injected in-house with a mixture of plasmids coding for the guide RNA and repair template DNA. In total, 100 potential candidate flies were screened exploiting restriction fragment-length polymorphism arising from the loss of a restriction digestion site introduced in parallel with the N595K mutation (*SI Appendix*, Fig. S3). We recovered 2 independent knockin lines (10.3 and 19.1) carrying the mutation, *sxc*^*N595K*^. Presence of the mutation was confirmed by sequencing the region ∼250 base pairs upstream and downstream from the mutation. Additionally, the lines were further validated by sequencing the full-length *sxc* mRNA. Both lines developed to adulthood without apparent homeotic defects. Transheterozygous *sxc*^*1*^*/sxc*^*H537A*^, *sxc*^1^/*sxc*^*N595K-19.1*^, and *sxc*^1^/*sxc*^*N595K-10.3*^ flies with presumably further reduced OGT activity were viable (*SI Appendix*, Fig. S4).

**Fig. 3.**
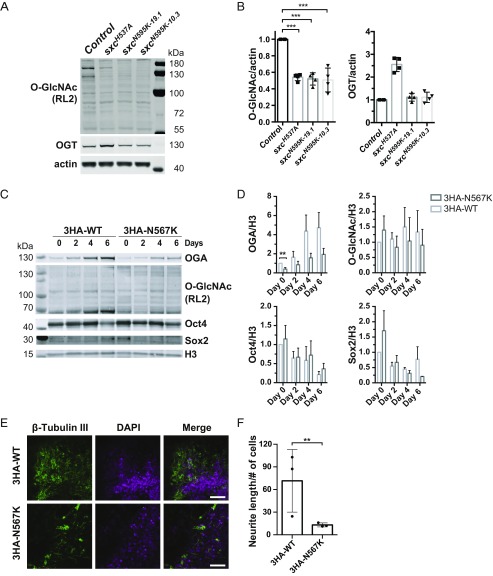
The N567K OGT mutation leads to altered O-GlcNAc homeostasis in *D. melanogaster* and neurite outgrowth defect in mESCs. (*A*) Immunoblot on 3- to 5-d-old adult *Drosophila* head lysates. (*B*) Quantification of O-GlcNAc modification on proteins and OGT protein level, normalized to actin signal. Protein O-GlcNAc modification is decreased in *sxc^N595K 19.1^* (***P < 0.0001) and *sxc^N595K 10.3^* (***P < 0.0001) lines to a comparable level found in the hypomorphic *sxc^H537A^* flies (***P < 0.0001). *n* = 4, mean ± SD. ANOVA with Tukey test. (*C*) Immunoblots on full cell lysate of 3HA-OGT^WT^ and 3HA-OGT^N567K^ mESCs showing OGA, Oct4, Sox2, Histone 3 (H3), and protein O-GlcNAcylation (RL2) levels. mESCs were differentiated for 0, 2, 4, and 6 d in N2B27 medium. (*D*) Quantification of immunoblots of OGA, Oct4, Sox2, and global protein O-GlcNAcylation in differentiated 3HA-OGT^WT^ and 3HA-OGT^N567K^ mESCs normalized to H3 signal. *n* = 4, mean ± SEM; **P = 0.012. Multiple *t* test using the Holm–Sidak method. (*E*) Immunofluorescence microscopy of 3HA-OGT^WT^ and 3HA-OGT^N567K^ mESCs after 8 d of neuronal differentiation in N2B27 medium. Cells were stained for tubulin III (green) and DAPI (magenta). (Scale bar, 50 μm.) (*F*) Quantification of neurite outgrowth. Total neurite length was measured per microscopic image by tracking β-tubulin III signal and then normalized to the number of nuclei (DAPI). (3HA-OGT^WT^, 108 to 800 nuclei per image; 3HA-OGT^N567K^, 260 to 740 nuclei per image) **P = 0.0079, Student *t* test, mean ± SD, *n* = 3.

To probe the effect of the N595K mutation on OGT enzymatic activity and stability in vivo, we subjected adult fly head and embryo lysates to Western blotting with an O-GlcNAc antibody, RL2 (Fig. 3*A* and *SI Appendix*, Fig. S5). O-GlcNAc levels were reduced to levels comparable to those in the hypomorph *sxc*^*H537A*^ flies (Fig. 3*A* and *SI Appendix*, Fig. S5). OGT protein levels are expressed at wild-type level (Fig. 3*A* and *SI Appendix*, Fig. S5). Taken together, phenotypic and molecular characterization of *sxc*^*N595K*^ *Drosophila* lines revealed that the mutation leads to reduction of protein O-GlcNAcylation.

### The N567K Mutation Leads to a mESC Neuronal Differentiation Phenotype.

The twins carrying the N567K OGT mutation, and the previously published male patients carrying OGT mutations in the TPR domain, all suffered from neurodevelopmental delay. We next explored the early events of neuronal development by studying effects on pluripotency and differentiation in mESCs, a cellular system amenable to genetic modification. We generated male mESC lines expressing C-terminal triple hemagglutinin (3HA) -tagged version of wild-type OGT (3HA-OGT^WT^) and carrying the N567K mutation (3HA-OGT^N567K^) using 2 rounds of CRISPR/Cas9-mediated gene editing at the endogenous locus (*SI Appendix*, Figs. S6 and S7). At each step, at least 2 clones with the correct genotype were isolated and selected for further analysis.

Next, we examined whether neuronal differentiation was altered in 3HA-OGT^N567K^ cells. Expression of pluripotency markers Sox2 and Oct4 was unaffected during the first 6 d of differentiation (Fig. 3 *C* and *D*). Neurite outgrowth was assessed after 8 d. Neurite length was significantly reduced in 3HA-OGT^N567K^ cells compared with 3HA-OGT^WT^ control cells immune-labeled against the neuron-specific β-tubulin III protein (Fig. 3 *E* and *F*). Thus, the N567K mutation leads to an mESC neuronal differentiation phenotype.

### The N567K Mutation Leads to Misprocessing of HCF1.

**Fig. 4.**
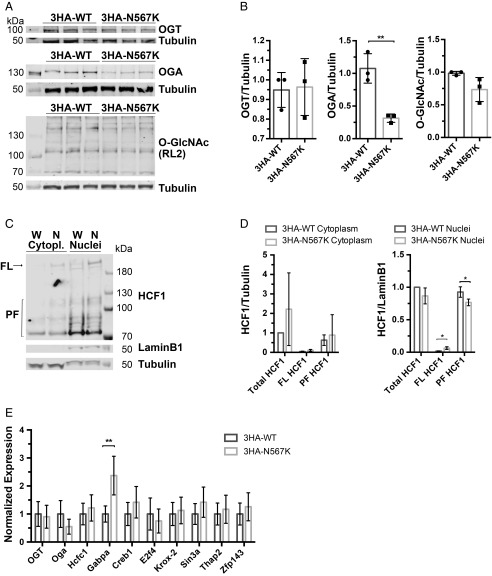
N567K mutation leads to defects in HCF1 processing and downstream gene expression in 3HA-OGT^N567K^ pluripotent mESCs. (*A*) Immunoblots showing OGT, OGA, and protein O-GlcNAcylation (RL2) levels in 3HA-OGT^WT^ and 3HA-OGT^N567K^ undifferentiated mESCs. (*B*) Quantification of immunoblots of global protein O-GlcNAcylation, OGA, and OGT protein level in undifferentiated mESCs normalized to tubulin signal. *n* = 3, mean ± SD. Unpaired *t* test. **P = 0.0049. (*C*) 3HA-OGT^N567K^ mESCs show impaired HCF1 proteolytic cleavage. Western blot analysis for HCF1, tubulin (cytoplasmic marker), and Laminin B1 (nuclear marker) indicates decreased level of proteolytic fragments of HCF1 in 3HA-OGT^N567K^ compared with 3HA-OGT^WT^ mESCs. (*D*) Quantification of immunoblots on nuclear and cytoplasmic fractions of undifferentiated 3HA-OGT^N567K^ mESCs compared with 3HA-OGT^WT^ mESCs control line showing HCF1 levels. HCF1 full-length (FL) and proteolytic fragments (PF) normalized to tubulin signal for the cytosolic fraction and to Lamin B1 signal for nuclear fraction. *n* = 3, mean ± SD. Multiple *t* test. *P = 0.0189 for FL and *P = 0.044 for PF. (*E*) qPCR analysis of gene expression of HCF1 targets in undifferentiated 3HA-OGT^N567K^ and 3HA-OGT^WT^ mESCs. Mean ± SEM, *n* = 3. **P = 0.002.

We next attempted to uncover the molecular mechanisms underpinning the observed differentiation phenotypes. We tested the levels of protein O-GlcNAcylation using Western blotting (Fig. 4*A*). Surprisingly, levels of protein O-GlcNAcylation in pluripotent mESCs appeared comparable in 3HA-OGT^N567K^ and 3HA-OGT^WT^ cells. Previous studies have shown that (an as yet to be discovered) feedback mechanism regulates OGA and OGT expression levels in response to protein O-GlcNAcylation levels (42, 43, 66, 67). Indeed, while OGT levels appeared to be unaltered, OGA levels were abrogated in 3HA-OGT^N567K^ pluripotent mESCs (Fig. 4 *A* and *B*), presumably to compensate for reduced OGT activity, but appeared normal during differentiation (Fig. 3 *C* and *D*).

Next, we investigated the effects of the N567K mutation on the posttranslational maturation and cellular localization of HCF1 in pluripotent mESCs. Western blot analysis on cytoplasmic and nuclear fractions revealed an elevated ratio of full-length versus total HCF1 in the nuclear fractions of 3HA-OGT^N567K^ compared with 3HA-OGT^WT^ mESCs, while the levels of HCF1 proteolytic products were reduced (Fig. 4 *C* and *D*). This is in agreement with the impaired OGT-mediated proteolytic cleavage of HCF1 observed in vitro (Fig. 2*C*). HCF1 contributes to the regulation of transcriptional programs associated with controlling cell cycle and pluripotency (68, 69). Interestingly, the expression of some of the HCF1 interactors are also regulated by HCF1 itself (51, 66, 69). As an initial attempt to uncover possible candidate genes affected by HCF1 misprocessing, we investigated the expression levels of known HCF1 partners by qRT-PCR (Fig. 4*E*). The Ets transcription factor GA-binding protein subunit α (GABPA), a component of the nuclear respiratory factor-2 (NRF2) complex, showed a 2.3-fold increase in mRNA level in 3HA-OGT^N567K^ cells compared with 3HA-OGT^WT^ control samples (*P* = 0.002). GABPA/NRF2 regulates mitochondrial biogenesis and cell cycle progression (70, 71). Taking these data together, the OGT N567K mutation leads to misprocessing of HCF1 in mESCs.

## Concluding Remarks

Previous studies have established a link between missense mutations in OGT and XLID in young males. These patients are hemizygous for the mutant OGT allele, with this genetic defect being present in all cells from early development to later life (41–43). However, residing exclusively in the OGT TPR domain, it was not clear whether these mutations led to the observed phenotypes through loss of glycosyltransferase activity, HCF1 misprocessing, or disruption of the OGT interactome (41–43). Here, we have presented female twins with XLID who are heterozygous for an N567K mutation in the OGT catalytic domain. Despite a wild-type *ogt* allele being present in all cells of these patients, its expression is arbitrarily silenced due to X chromosome inactivation at the late blastocyst stage of embryogenesis to compensate for gene dosage (72). Thus, these females represent an example of mosaic expression of an OGT XLID variant causing developmental abnormalities. Previous examples of female carriers of X-linked genetic disorders showed correlation between the X inactivation pattern and the severity of the condition (73–75). Hence, it is possible that the different etiology of ID in twins is the consequence of distinct spatial patterns and skew of X-inactivation.

The N567K variant of OGT retains negligible levels of catalytic activity. Thus, a possible mechanism underpinning the XLID phenotype is reduced protein O-GlcNAcylation on a subset of OGT target proteins at specific time points of development or differentiation. Previous genetic studies in mice have highlighted the vital role of OGT in both neuronal development and adult life (21, 33, 37–39). OGT is capable of transferring GlcNAc onto over 4,000 different substrate proteins; it possesses an acceptor-peptide specificity with a preference to a degenerated sequon motif (57, 76, 77). Our structural analysis has uncovered that the N567K mutation changes the surface of the substrate binding pocket, thereby altering substrate binding ability of the N567K OGT. Previously reported XLID associated mutations lie in the TPR domain of OGT (40–43, 78), so potentially influencing its scaffolding function or reducing O-GlcNAcylation on an as yet unknown, subset of substrates. The N567K mutation may affect the O-GlcNAc proteome globally yet leads to a similar clinical phenotype, suggesting it is the catalytic activity/dosage of OGT that is important.

We have identified one of the OGT substrates, HCF1, as a potential candidate that could at least partially explain some of the OGT N567K patient phenotypes. We showed that O-GlcNAcylation and proteolytic activation of HCF1 is significantly decreased, including in mESC nuclei. The HCF1 protein plays an important role in cell growth and cell cycle control through interacting with transcriptional complexes and epigenetic regulators (79–83). The HCF1 gene itself has been identified as an intellectual disability gene causing cobalamin type X deficiency, craniofacial abnormalities, and prenatal onset of microcephaly in the most extreme cases (52, 53, 84). Less-severe HCF1 mutations have been associated with ID and autism spectrum disorder without cobalamin deficiency (54). Patients with N567K OGT variant did not suffer from cobalamin deficiency and expressed a much milder form of congenital anomalies than the severe HCF1 group, suggesting that a degree of HCF1 activity is retained. Interestingly, we observed an increase in full-length HCF1 protein level and decreased level of the mature proteolytic fragment in nuclei, showing that HCF1 processing is affected *in cellulo*, albeit with retention of some activity. Surprisingly, the expression of the transcriptional factor GABPA downstream of HCF1 was increased indicating that the N567K mutation in OGT could translate into moderate molecular changes downstream of HCF1. The opposite effect, decreased expression of GABPA, was detected upon overexpression of the HCF1 N-terminal fragment (HCF_N_) (68). GABPA belongs to the E-26 family of DNA binding factors and regulates expression of several proteins required for mitochondrial DNA transcription and replication (85). Moreover, GABPA modulates the expression of genes involved in cell cycle control, apoptosis, differentiation, and hormonal regulation. Its importance in early embryonic development was demonstrated in knockout mouse studies (86). However, there is no established link between GABPA and neurodevelopment. Furthermore, expression of several other transcriptional factors targeted by HCF1—such as *Creb1*, *E2F4*, *Krox-2*, *Sin3a*, *Thap2*, and *Zfp143*—were found unaltered, suggesting that partial abrogation of HCF1 cleavage may affect just a small subset of targets, whose expression is influenced by the altered ratio of proteolytically cleaved and uncleaved HCF1.

While OGT is critical for mESC maintenance of pluripotency and differentiation, we have detected no difference at the level of pluripotency markers, Sox2 and Oct4, between 3HA-OGT^WT^ and 3HA-OGT^N567K^ mESCs. The pluripotency factor Sox-2 is O-GlcNAc modified at Ser248 (87) and this posttranslational modification is indispensable for sustaining pluripotency. However, its O-GlcNAc modification is not required for differentiation, with cells expressing an O-GlcNAc–deficient mutant version of Sox2 exhibited enhanced reprogramming ability (87). Furthermore, mESCs expressing O-GlcNAc–deficient Sox2 displayed changes in their transcriptional network, specifically increasing the expression of genes vital to maintain pluripotency (87). It is feasible that a similar mechanism is responsible to compensate for pluripotency defects induced by the N567K OGT mutation.

A *Drosophila* model of the N567K OGT mutation showed reduced protein O-GlcNAcylation. However, the abundance of O-GlcNAc–modified proteins was similar in 3HA-OGT^WT^ and 3HA-OGT^N567K^ mESCs. Interestingly, we observed decreased OGA protein levels as a compensatory regulatory mechanism responsible for maintaining protein O-GlcNAc levels in undifferentiated mESCs. Our finding is in line with previous data reported on patient-derived cell lines carrying XLID mutations in the TPR domain of OGT (42, 43). Although there is no evidence for a link between OGA and XLID, given the role of OGA in regulating transcription (88–91), it cannot be excluded that changes in OGA levels directly contribute to the XLID phenotype.

In agreement with previous studies implying a requirement for OGT function in normal neuron differentiation, morphology, and health (27, 37, 92–94), abnormal OGT activity in 3HA-OGT^N567K^ cells resulted in reduced neurite outgrowth. These data suggest a possible mechanistic link between the mutation and the microcephaly and neurodevelopmental cognitive impairments observed in the patients.

In summary, we have shown that impaired catalytic activity of OGT leads to neurodevelopmental defects in humans, through combined downstream effects of impaired OGT activity and reduced HCF1 proteolytic processing. Further studies are required to define the mechanisms downstream of impaired OGT catalytic activity that affect neurodevelopment resulting in intellectual disability.

## Materials and Methods

The study was approved by the University of Dundee Institutional Risk Assessment committee, and 1 of each participants’ parents provided written, informed consent. The DDD study has UK Research Ethics Committee (REC) approval (10/H0305/83, granted by the Cambridge South Research Ethic Committee, and GEN/284/12 granted by the Republic of Ireland REC).

### X-Inactivation Analysis.

To determine if X chromosome inactivation occurred randomly or nonrandomly, the ratio of methylated and unmethylated maternal and paternal X chromosomes was determined. (*SI Appendix*, Fig. S1). Sites are methylated on the inactive X chromosome and unmethylated on the active X chromosome. The method utilizes the androgen receptor sequence that is encoded on the X chromosome (Xq11.2-q12) and contains a highly polymorphic polyglutamine (CAG) trinucleotide repeat in exon 1. There are 20 alleles of androgen receptor, with 11 to 31 repeats reported in the general population and 85 to 90% of patients are heterozygous for this locus, thus suitable for X-inactivation analysis. For experimental procedure, see *SI Appendix*, *SI Materials and Methods*.

### Structure Solution.

Crystallization of truncated OGT_WT/N567K_ (residues 323–1044) was performed as described previously (57) (for protein expression, purification, and crystallization, see *SI Appendix*, *SI Materials and Methods*). The structure was solved by molecular replacement using the structure for OGT_WT_ [PDB ID code 5C1D (57)] as the search model. The resulting model was manually refined using Coot (95) and REFMAC (96), respectively. The editing and refinement of the model was iterated until it was in complete agreement with the data. Scaling and model building statistics can be seen in Table 2.

### Enzyme Activity Assays.

Michaelis–Menten kinetics of OGT were measured using a fluorometric assay, as described previously (97), with the exception of reduced reaction volume of 25 µL and usage of 384-well plate. As acceptor substrate, P1 (KKVPVSRA) and P2 (KKVAVSRA) were used. Additional O-GlcNAcylation assays were performed on TAB1 protein (residues 7–420). Proteolytic assays were performed as described previously (43), except a noncleavable HCF1-rep1 fragment was used instead of OGA treatment as the negative control. For further details, see *SI Appendix*, *SI Materials and Methods*.

### mESC Culture.

mESC AW2 line was acquired from the MRC Centre for Regenerative Medicine, Institute for Stem Cell Research, University of Edinburgh (98). mESCs were cultured in an undifferentiated state in 0.1% gelatin (wt/vol)-coated plates in GMEM BHK-21 supplemented with 10% FBS (vol/vol), 0.1 mM MEM nonessential amino acids, 1 mM sodium pyruvate, 0.1 mM 2-mercaptoethanol, and 100 units/mL LIF at 37 °C in 5% CO_2_. For differentiation assays, cells were cultured following a previously published protocol (99). Briefly, cells were cultured in DMEM and F12 media mixed at 1:1 ratio (vol/vol), supplemented with modified N2 (25 mg/mL insulin, 100 μg/mL apo-transferrin, 6 ng/mL progesterone, 16 μg/mL putrescine, 30 nM sodium selenite, and 50 μg/mL BSA fraction) and neurobasal supplement B27. Medium was renewed every 2 d. For generation of CRISPR/Cas9 lines, Immunocytochemistry, Western blotting, and qPCR analyses details are provided in *SI Appendix*, *SI Materials and Methods*.

### *Drosophila* Husbandry, Microinjection, and Genetics.

Fly stocks were maintained on Nutri-Fly Bloomington Formulation fly food at 25 °C. Vasa::Cas9 (#51323), *sxc*^*1*^*,bw*^*1*^*,sp*^*1*^*/SM5* (#3058) and *Df(2R)BSC630/CyO* (#25705) stocks were obtained from the Bloomington *Drosophila* Stock Centre. The *sxc* hypomorphic allele, *sxc*^*H537A*^, was described in ref. 63. The *sxc*^*N595K*^ *Drosophila* lines were generated by CRISPR/Cas9 mutagenesis, as described in *SI Appendix*, *SI Materials and Methods*.

### Western Blotting from *Drosophila* Samples.

Sample preparation of embryos and fly heads and Western blotting were performed as described in *SI Appendix*, *SI Materials and Methods*.

## Supplementary Material

Supplementary File
