# Supplementary material for: Catalytic deficiency of O-GlcNAc transferase leads to X-linked intellectual disability
Source: Proc Natl Acad Sci U S A. 2019 Jul 11;116(30):14961–70. doi: 10.1073/pnas.1900065116 (PMC6660750; doi:10.1073/pnas.1900065116)
Supplement: Supplementary File [file pnas.1900065116.sapp.pdf]

SI Appendix

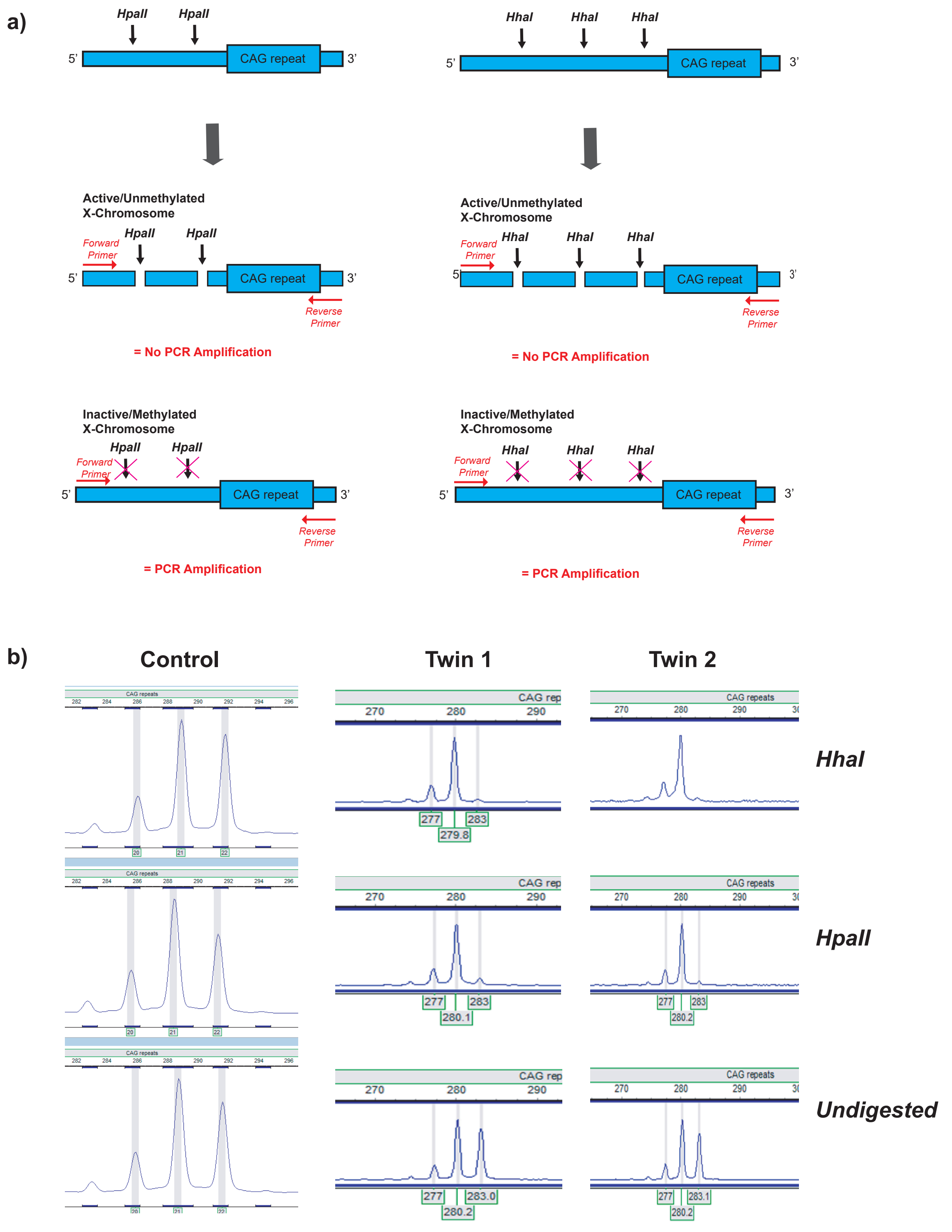

www.pnas.org/cgi/doi/10.1073/pnas.1900065116

c)

Patient: Twin 1

Conclusion/Interpretation: **Severely skewed X-inactivation**

Allele Size	Ratio	Interpretation
280	0.93	Severe skewing. The X chromosome with the 283 allele is preferentially active. Unable to identify X chromosome with disease-causing variant.
283	0.07	

NAME	Size #1	Area #1	Size #2	Area #2	Size #3	Area #3	AlleleCount	RatioRAW	RatioAdjust	RatioFinal	ratios are relative to smallest (true) allele
CN_X_inact_Hh_G10.fsa	276.9	489	279.8	1825	282.6	94	3	0.934	0.967	0.93	
CN_X_inact_Hp_E11.fsa	277.3	1542	280.1	5415	282.9	668	3	0.852	0.885		
CN_X_inact_un_C12.fsa	277.2	1290	280.2	5150	283	4406	3	0.466			

Patient: Twin 2

Conclusion/Interpretation: **Severely skewed X-inactivation. HhaI digest fail**

Allele Size	Ratio	Interpretation
280	0.93	Severe skewing. The X chromosome with the 283 allele is preferentially active. Unable to identify X chromosome with disease-causing variant.
283	0.07	

NAME	Size #1	Area #1	Size #2	Area #2	Size #3	Area #3	AlleleCount	RatioRAW	RatioAdjust	RatioFinal	ratios are relative to smallest (true) allele
GN_X_inact_Hh_F10.fsa							0	UNINFORMATIVE		0.93	
GN_X_inact_Hp_D11.fsa	277.5	1857	280.2	6935	283	454	3	0.917	0.928		
GN_X_inact_un_B12.fsa	277.5	1658	280.2	6398	283.1	4952	3	0.489			

d)

Average skewing (Ratio)	Interpretation
0.50-.074	Within range seen in normal patients
≥0.75-0.79	Borderline skewing
≥0.80-0.89	Skewed
≥0.90-0.99	Severe skewing
1	Complete skewing

Supplementary Figure S1: X-inactivation analysis.

(a) Schematic representation of the methodology. Each sample is digested separately with two enzymes: *HhaI* (3 cut sites) and *HpaII* (2 cut sites). These sites are expected to be methylated on the inactive X chromosome and unmethylated on the active X chromosome. Both enzymes are methylation sensitive and will not cut at methylated sites.

(b) Analysis of the PCR products. Peak 280 and 283 represents the PCR products of maternal or paternal allele.

(c) Table shows the results of X-inactivation analysis for Twin 1 and Twin 2. The ratios were calculated as Area #1 / (Area #1 + Area #2) = RatioRAW; ((Enzyme RatioRAW value – 0.5) – (Undigested RatioRAW value – 0.5) + 0.5) = RatioAdjust (pref. amp. correction); (RatioAdjust HhaI value + RatioAdjust HpaII value) / 2 = RatioFinal (i.e. average from both enzymes).

(d) Table shows the categorical interpretation of data from X-inactivation analysis.

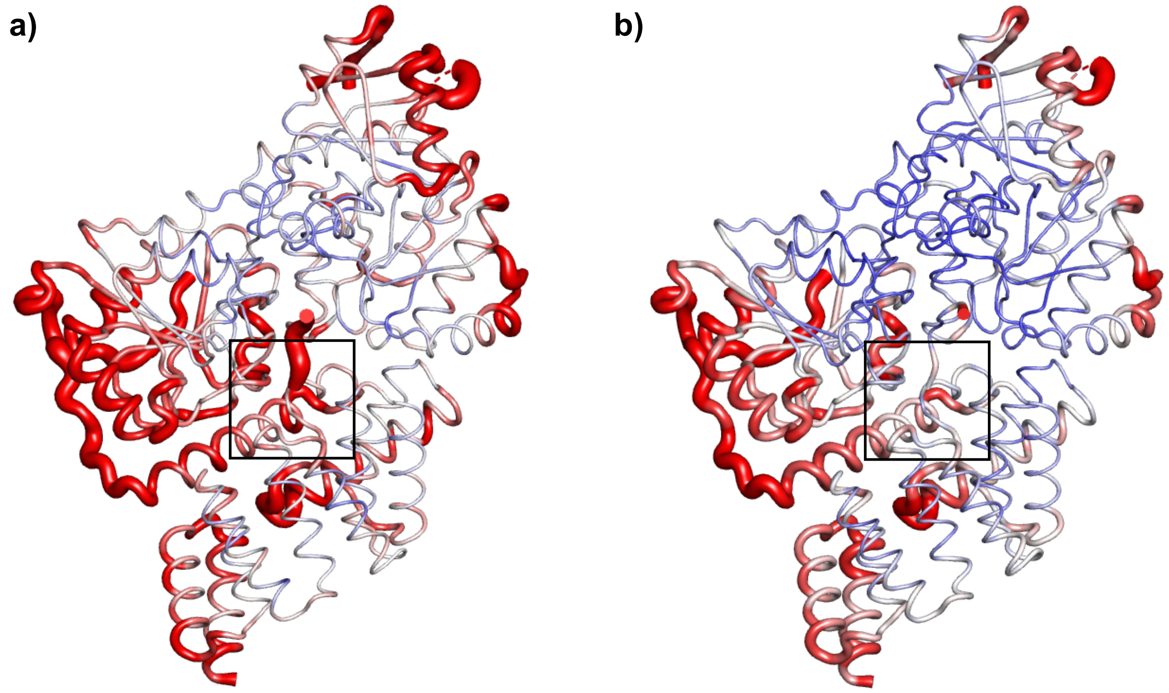

Supplementary Figure S2: Structural analysis of OGT_{N567K} *versus* OGT_{WT} ternary complexes.

(a) Entire OGT_{N567K}:P2 complex (PDB: 6IBO) shown in putty representation with the loop colour and radius both highlighted as a function of B-factors, with red colour and large loop radius indicating high B-factors. Acceptor peptide is highlighted in black square.

(b) Same as in (**a**), but showing OGT_{WT}:RB2 complex (PDB: 5C1D, Pathak et al., 2015). RB2: Retinoblastoma-like protein 2.

Hinfl

WT TAGGTTATCTTAGCTCGGACTTCGGGAATCATCCTACCTCGCAT

I G Y L S S D F G N H P T S H

sxc^{N595K} TAGGTTATCTTAGCTCCGATTTTGGCAAACATCCTACCTCGCAT

I G Y L S S D F G K H P T S H

Supplementary Figure S3: Genomic DNA sequence of *Drosophila melanogaster* wild type and N595K *sxc* gene and translated protein product.

Modification highlighted in orange is responsible for the N595K mutation, nucleotides highlighted in green are silent wobble sites. *HinfI* restriction site is lost in *sxc*^{N595K} flies.

Parental cross	%CyO	%non CyO	expected ratio if no lethality occurs	Total
***sxc**^{N595K-10.3}*/**CyO** x ***sxc**^{N595K-10.3}*/**CyO**	69	31	66% CyO vs 33% non-CyO	870
$sxc^{N595K-19.1}$/CyO x $sxc^{N595K-19.1}$/CyO	76	24	66% CyO vs 33% non-CyO	182
$sxc^{N595K-10.3}$/CyO x sxc^{1}/CyO	54	46	66% CyO vs 33% non-CyO	334
$sxc^{N595K-19.1}$/ $sxc^{N595K-19.1}$ x sxc^{1}/CyO	43	57	50% CyO vs 50% non-CyO	310
$sxc^{N595K-10.3}$/CyO x *Df(2R)BSC630*/CyO	59	41	66% CyO vs 33% non-CyO	611
$sxc^{N595K-19.1}$/ $sxc^{N595K-19.1}$ x *Df(2R)BSC630*/CyO	55	45	50% CyO vs 50% non-CyO	174
$sxc^{N595K-10.3}$/CyO x sxc^{H537A}/sxc^{H537A}	45	55	50% CyO vs 50% non-CyO	277
$sxc^{N595K-19.1}$/ $sxc^{N595K-19.1}$ x sxc^{H537A}/sxc^{H537A}	0	100	CyO not expected	207

Supplementary Figure S4: Viability during development is not compromised in $sxc^{N595K-10.3}$ and $sxc^{N595K-19.1}$ *Drosophila* lines.

$sxc^{N595K-10.3}$ and $sxc^{N595K-19.1}$ *Drosophila* develops to adulthood normally and viability during development is not affected by the presence of sxc^{N595K} mutation. Further manipulation of O-GlcNAc levels by reduced DmOGT function *(*sxc^{1} *or Df(2R)BSC630* alleles) showed an impact on neither development nor survival.

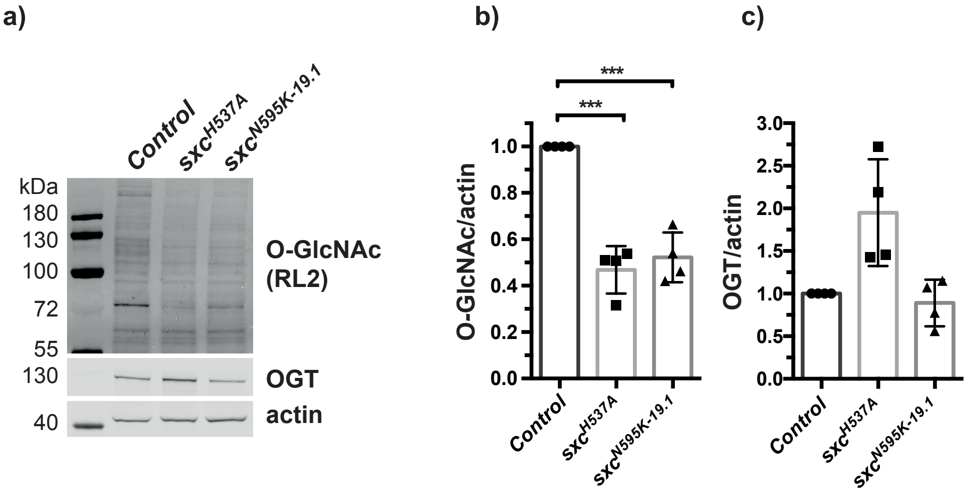

Supplementary Figure S5: Quantification of protein O-GlcNAcylation and OGT protein level in *sxc*$^{N595K\text{-}19.1}$ *Drosophila* embryo samples.

(a) Immunoblot on *Drosophila* embryo lysates.

(b) Quantification of O-GlcNAc modification on proteins, normalized to actin signal. Protein O-GlcNAc modification is decreased in *sxc*$^{N595K\text{-}19.1}$ (***$p < 0.0001$), however, comparable to the level found in the hypomorph *sxc*^{H537A} *Drosophila* embryos. n = 4, mean ± SD. ANOVA with Tukey test.

(c) Quantification of DmOGT protein level normalized to actin signal. OGT protein level was detected at Control level in *sxc*$^{N595K\text{-}19.1}$. n = 4, mean ± SD. ANOVA with Tukey test.

BfmI *PstI*

WT GAGCGACTTTATCTGCAGATGTGGGAGCATTATGCAGCTGGCAAC

E R L Y L Q M W E H Y A A G N

AAACCTGACCACATGATTAAGCCTGTTGAAGTCACCGAGTCAGCC

K P D H M I K P V E V T E S A

TGAATAAAGACTGCGCACAGGAGAATTGCCCTATACC

*** I K T A H R R I A L Y L**

3HA GAGCGACTGTACCTCCAAATGTGGGAGCATTATGCAGCTGGCAAC

E R L Y L Q M W E H Y A A G N

AAACCTGACCACATGATTAAGCCTGTTGAAGTCACCGAGTCAGCC

K P D H M I K P V E V T E S A

TCTAGATACCCATACGATGTTCCTGACTATGCGGGCTATCCCTAT

S R Y P Y D V P D Y A G Y P Y

GACGTCCCGGACTATGCAGGATCCTATCCATATGACGTTCCAGAT

D V P D Y A G S Y P Y D V P D

TACGCTTGAATAAAGACTGCGCACAGGAGAATTGCCCTATACC

Y A * I K T A H R R I A L Y L

Supplementary Figure S6: Gene-editing of mESCs to introduce 3HA-tag into the endogenous OGT gene.

Genomic DNA sequence of mouse wild type and 3HA OGT gene and translated protein product is shown. *BfmI* and *PstI* restriction sites are lost in 3HA cells. Modification highlighted in green are silent mutations, nucleotides highlighted in blue code for 3HA-tag.

WT

CTCAGTGATGGCCGATTGCGTGTAGGCTATGTGAGTTCTGACTTC
L S D G R L R V G Y V S S D F

HinfI

GGGAATCACCCTACTTCACACCTTATGCAGTCTATTCCAGGCATG
G N H P T S H L M Q S I P G M

N567K

CTCAGTGATGGACGCCTGCGCGTGGGCTATGTGAGTTCTGACTTC
L S D G R L R V G Y V S S D F

GGGAAGCACCCTACTTCACACCTTATGCAAAGCATCCCAGGCATG
G K H P T S H L M Q S I P G M

Supplementary Figure S7: Gene-editing of mESCs to introduce N567K mutation into endogenous OGT gene.

Genomic DNA sequence of mouse wild type and N567K OGT gene and translated protein product is shown. Modification highlighted in orange is responsible for the N567K mutation, nucleotides highlighted in green are silent wobble sites. *HinfI* restriction site is lost in N567K cells.

List of Primers	Sequence (5'->3')
Actb Forward	GATCAAGATCATTGCTCCTCCTG
Actb Reverse	CAGCTCAGTAACAGTCCGCC
Gapdh Forward	ACCCTTAAGAGGGATGCTGC
Gapdh Reverse	GGGACGAGGAAACACTCTCC
Pgk1 Forward	GCTATCTTGGGAGGCGCTAA
Pgk1 Reverse	AAAGGCCATTCCACCACCAA
OGT Forward	CCCCCTGAGCCCTTCAAAAC
OGT Reverse	TCGTTGGTTCTGTACTGTCGG
OGA Forward	TGCAGTGGTTAGGGTGTCG
OGA Reverse	AGCAAACGCTGGAACTCTCC
E2F4 Forward	TGCAGATGCTTTGCTGGAGA
E2F4 Reverse	CTAGCAGCACCTCGATAGGC
Sin3 Forward	AACTCAGATGACCCCGTGGA
Sin3 Reverse	GATACGCCGCAAATTCCTTGG
HCF1 Forward	GTGACCCAGGTGGTGCTAAA
HCF1 Reverse	TTGACAGCAGAGACGGTGAC
Krox-2 Forward	TGGCAGGACTAGCAGGATTC
Krox-2 Reverse	CCCCTCTGCTCCCTGTAGAT
Zfp143 Forward	GCAGCGTTCCATACAGCATC
Zfp143 Reverse	TCTCCAAGCTGAACTGCGAT
Gabpa Forward	CCGGGGAACAGAACAGGAAA
Gabpa Reverse	CAGTCTCGAGCATCCTTGTCA
Creb3 Forward	GAAAGCGGAGATTTGTGGGC
Creb3 Reverse	TTGCACGGAGTTCTCGGAAG
THAPII Forward	ACATCAGCTTCCACAGGTTTCC
THAPII Reverse	AGTCGTCGGGTTTGTCCTGT

Supplementary Table S1: Primers used for RT-qPCR analysis of expression level of HCF1 target genes.

**Guide RNA**	gRNA_sxcT595_F	GTCGTTATCTTAGCTCGGACTTC
**Guide RNA**	gRNA_sxcT595_R	AAACGAAGTCCGAGCTAAGATAA
**Genomic DNA**	T59fix_BAM_F	AAAGGATCCAGTTAAAGGTTTTATGGAAAAGGTACG
**Genomic DNA**	T59fix_NOT_R	TTTGCGGCCGCAAAAAATTATATTTACCAAAGTCACTGACGG
**Mutagenesis**	BA_N595K_GLAS_F	CTTCGAATAGGTTATCTTAGCTCCGATTTTGGCAAACATCCTACCTCGCATTTAATGCAATC
**Mutagenesis**	BA_N595K_GLAShelp_R	GTTACAGAATCCGTAATAATGTAATCC
**Genotyping**	T595_DIG F	ATTCAGTCTTACCGAACGGCTCTAAAG
**Genotyping**	T595_DIG R	GACTCTCGACTGATTTTGTGTCGAAATG
**Genotyping**	T595_DIG_SEQ	CCGTTTACCATCCGTGCATC
**Line check**	T595_seq1	CAAGGCAAATTAAAGGAAGCG
**Line check**	T595_seq2	ATCTGTGCCAGGTCTTCATG
**Line check**	T595_oob F	AGTTGAAATGTACAAGCAACGTCAGAAC
**Line check**	T595_oob R	TGGAAAATATTACTCTATCAGGAGATATTC
**cDNA check**	DmOGT_examine_F	ATGCATGTTGAACAAACACGAATAAATATG
**cDNA check**	DmOGT_examine_R	TTA TACTGCTGAAATGTGGTCCGGAAG
**cDNA check**	dOGT_seq700R	CAACCAAATTTCTCCCTGAGC
**cDNA check**	dOGT_seq500F	TGCATTAGTTGCTGCTCGTG
**cDNA check**	dOGT_seq1000F	ATCTTGCAAACGCTCTCAAAG
**cDNA check**	dOGT_seq1500F	ATGCATACTGCAATTTGGCTC
**cDNA check**	dOGT_seq2050F	CTTCGCCCTGCTCCTATTC
**cDNA check**	dOGT_seq2600F	CTGTTGTGTATTGCAATTTTAATC

Supplementary Table S2: Primers used for generating and genotyping *sxc*^{N595K} Drosophila lines.

**Guide RNA**	L3HA_F	CACCGTAATTCCATTGTGTATTGTT
**Guide RNA**	L3HA_R	AAACAACAATACACAATGGAATTAC
**Guide RNA**	3HAR_F	CACCGAGCGACTTTATCTGCAGATG
**Guide RNA**	3HAR_L	AAACCATCTGCAGATAAAGTCGCTC
**Genomic DNA**	MmOGT_HA_F	AAAGGATCCAGGCAGGAGGATTGCCTTAAGTTTTAGG
**Genomic DNA**	MmOGT_HA_R	AAAGCGGCCGCGAAAGAAATGGGAAGAACAGGACTC AC
**Sequencing**	MmOGT_HA_seq1	GAAAGTGTCCCACAGACATG
**Sequencing**	MmOGT_HA_seq2	GAATCTCTTAGAGTTTTGCAGC
**Mutagenic GB***	MmOGT_HA_GB	ctgatttgtaaatttggttctctttgtttttaccacctagcctgaagaaaattcgtggca aagtctggaaacagagaatatctagccctctgttcaacacAaaGcaGtaTac CatggaattagagcgactGtaCctCcaAatgtgggagcattatgcagctggc aacaaacctgaccacatgattaagcctgttgaagtcaccgagtcagccTCTA GATACCCATACGATGTTCCTGACTATGCGGGCTATCC CTATGACGTCCCGGACTATGCAGGATCCTATCCATAT GACGTTCCAGATTACGCTtgaataaagactgcgcacaggagaatt gccctatacctgagcctcaaccttctgggggaagggaactagataacatgcttt gtgtgtatctgtgtagttctgtgttgcag
**Mutagenesis**	3xHA_fwd	CTGATTTGTAAATTTGGTTCTCTTTG
**Mutagenesis**	3xHA_rev	CTGCAACACAGAACTACACAGATAC
**Genotyping**	3HAdiag_F	CTGGCCTGGGTCATTCCTCATTAATC
**Genotyping**	3HAdiag_R	CAATTCTCCTGTGCGCAGTCTTTATTC

Supplementary Table S3: Primers and gene-block used for introducing 3HA-tag to OGT gene and genotyping candidate 3HA-OGT^{WT} mES cell line.

* - upper case letters denote introduced changes in gene-blocks (GB).

**Guide RNA**	Glas_mGLE_F	CACCGCACCTTATGCAGTCTATTCC
**Guide RNA**	Glas_mGLE_R	AAACGGAATAGACTGCATAAGGTGC
**Guide RNA**	Glas_mGRI_F	CACCGTCACATAGCCTACACGCAAT
**Guide RNA**	Glas_mGRI_R	AAACATTGCGTGTAGGCTATGTGAC
**Genomic DNA**	Mouse_Glas_BclI_F	AAATGATCAAGGGTTTTCCTACTCCATTCACATTTC
**Genomic DNA**	Mouse_Glas_NotI_R	AAAGCGGCCGCCACCCTACAAAAGAATGAAACAACAAAGC
**Sequencing**	Mouse_Glas_seq1	GCACCAACATGCTCATGTAG
**Sequencing**	Mouse_Glas_seq2	GGTGATGGCGGAAGCCAATC
**Mutagenic GB***	MouseGlaWob_GB	gattaatgtccttcataaaccaccatatgaacatccaaaagacttgaagctcagtgatggAcgCCtgcgCgtGggctatgtgagttctgacttcgggaaGcaccctacttcacaccttatgcaAAGCatCccaggcatgcataatcctgataagtttgagg
**Mutagenesis**	MGW_F	CCAAAAGACTTGAAGCTCAGTGATGG
**Mutagenesis**	MGW_R	CTTATCAGGATTATGCATGCCTGG
**Genotyping**	MouseGlaDiag_F	CCTTTATATTTCTACCTCACAATTACAGG
**Genotyping**	MouseGlaDiag_R	GGAGGGGACAGTGTGCTCAATAACC

Supplementary Table S4: Primers used for introducing N567K mutation into OGT gene and genotyping candidate 3HA-OGT^{N567K} mES cell lines.

* - upper case letters denote introduced changes in gene-blocks (GB).

SI Appendix_SI Materials and Methods

X-inactivation analysis

DNA (200 ng) was digested with methylation-sensitive restriction enzymes followed by PCR for the CAG repeat in exon 1 of the AR gene. Each sample was digested separately with two methylation sensitive restriction enzymes: *HhaI* (3 cut sites) and *HpaII* (2 cut sites), and an undigested sample was used as control. PCR primers flanking the entire CAG repeat region including these restriction sites were used to amplify the CAG repeat sequence. PCR amplification occurred only on the inactive X chromosome where the methylation sensitive restriction enzymes were unable to cut the DNA. Intensities of digested and undigested DNA were compared in order to assess the X-inactivation pattern. Ratios and results are reported in **Supplementary Fig. S1**.

Protein expression and purification

Full length and truncated (residues 323-1044) OGT constructs were expressed in *E. coli* BL21(DE3) RIPL cells as N-terminal His and GST fusion proteins, respectively, as described previously (1). Briefly, transformed *E. coli* cells were grown in LB broth at 37 °C with agitation until OD_{600} reached 0.8, at which point the temperature was lowered to 18 °C and expression was induced by addition of 100 µM IPTG. Cells were lysed using French Press in base buffer (0.1 M Tris-HCl, pH 7.5, 0.15 M NaCl, 0.5 mM TCEP) supplemented with 25 mM imidazole 0.1 mg/ml DNase I and protease inhibitor cocktail (1 mM benzamidine, 0.2 mM PMSF, 5 mM leupeptin). For purification of GST-tagged constructs, imidazole was omitted from the base buffer. Then, recombinant proteins were affinity purified following manufacturers' guidelines. After cleavage of affinity tags, and negative pull-down using fresh affinity resin, dialyzed protein was loaded onto 5 ml HiTrap Q Sepharose FF anion exchange resin (GE Healthcare) and eluted with a linear gradient up to 60% of buffer B (0.1 M Tris-HCl, pH 8.5, 500 mM NaCl). Peak fractions were pooled, concentrated and further purified via size exclusion chromatography using 300 ml prepacked Superdex™ 200 column (GE Healthcare) equilibrated with base buffer. The peak fractions were concentrated to 10 mg/ml. For crystallisation,

truncated OGT was used fresh at 7 mg/ml concentration. For all other purposes, proteins were concentrated to 10 mg/ml, mixed 1:1 with 50% glycerol, snap-frozen and stored at -80˚C until use.

Protein crystallisation

Briefly, experiments were performed at 22 ºC using 24-well hanging drop crystallization plates, by combining 1 µl drops containing 7 mg/ml $OGT_{WT/N567K}$ (in base buffer), 3 mM UDP-5S-GlcNAc and 3 mM peptide with 2 µl of reservoir solution (1.45 M K_2HPO_4, 8 mM EDTA and 1% xylitol). Large, clear-faced crystals appeared overnight. Prior to diffraction experiments, individual crystals were cryo-protected in reservoir solution supplemented with 3.5 M maleic acid and flash-frozen in liquid nitrogen. Diffraction data were collected at the European Synchrotron Radiation Facility beamline ID23. Data were processed with XDS (2) and scaled to 2.23 Å using SCALA (3).

Enzyme activity assays

Reactions for Michaelis-Menten kinetics contained 0-254 µM acceptor peptide substrate, 200 µM UDP-GlcNAc and 50 nM OGT in 50 mM HEPES-NaOH pH 7.5, 0.1 mg/ml BSA and 10 µM $Na_2S_2O_4$. Reactions were stopped before 10% of the acceptor substrate was depleted by addition of 50 µl detection reagent (25 mM HEPES-NaOH pH 7.5, 10 mM NaCl, 15 µM xanthene based Zn(II) complex, 75 µM pyrocathecol violet and 50% methanol). The fluorescence was read using excitation and emission wavelengths of 485 nm and 530 nm, respectively. Data were background corrected and analysed using GraphPad Prism.

For the TAB1 *O*-GlcNAcylation assays TAB1 (1 µM) was combined with full length OGT_{WT} or OGT_{N567K} (0.1 µM) in the presence of 100 µM UDP-GlcNAc. The reaction mixtures were incubated ad 37 ºC from 2 to 50 minutes and subsequently stopped by addition of LDS loading buffer (4x, Thermo Fisher Scientific). Proteins were resolved by SDS-PAGE (4-12% acrylamide, Life Technologies) and transferred onto nitrocellulose membranes (GE Healthcare). After antibody treatment, progress of the reaction was visualised using LI-COR Odyssey scanner and associated quantification software.

Proteolytic assays was performed using HCF1-rep1 fragment (residues 867-1071) with GST and His tags at the N-terminus and C-terminus, respectively. HCF1-rep1 (2.5 µM) was combined with full length OGT_{WT} or OGT_{N567K} (1 µM) in the presence of 1 mM UDP-GlcNAc. Reaction mixtures were incubated at 37 ˚C for 2-8 h with gentle agitation. Reactions were stopped by addition of LDS loading buffer (4X) (Thermo Fisher Scientific). Proteins were resolved by SDS-PAGE (4-12% acrylamide, Life Technologies) and transferred onto nitrocellulose membranes (GE Healthcare). After antibody treatment, progress of the reaction was visualised using LI-COR Odyssey scanner and associated quantification software.

Generation of 3HA-OGT^{WT} and 3HA-OGT^{N567K} mES cell lines

For 3HA-OGT^{WT} mES cell line generation, we transfected wild type male AW2 mES cells with pBABED puro U6 and pX335 (Cas9 D10A) vectors containing the gRNA sequences selected using WTSI Genome Editing (4). Based on the regions recognized by the two gRNA sequences, silent mutations were designed in addition to the intended mutations, removing a *Pst*I and *Bfm*I restriction sites. A geneblock containing these changes was obtained from IDT (International DNA technologies). The geneblock was introduced into the cloned 2 kb region by restriction free cloning (5) and confirmed by DNA sequencing (5). For targeting, $2x10^5$ cells were seeded onto gelatine coated 24-well plate and transfected using 1 µg of vectors in a 1:1:1 ratio and Lipofectamine 3000 according to manufacturer's instructions. After 24 h, media was replaced and puromycin (3 µg/mL) was added to the cells for selection of 48 hours. Then the cells ware re-plated into 10 cm plates for recovery. Single cell selection was carried out using limited dilution according to 0.3 cell per well. For mutation identification restriction enzymes and genomic DNA sequencing analysis were used for mutation identification. For the restriction fragment length polymorphism assay 3HADiag_F and 3HADiag_R primers were used to amplify by PCR the mutated site and the silent mutation which eliminates a *Pst*I restriction site (**Supplementary Table S3**). The size of the PCR product (546 bp vs 450 bp for wild type) was used to screen for successful integration. To further confirm, the PCR product was then digested using *Pst*I or *Bfm*I. Clones negative for the restriction enzyme assay were

then sequenced to confirm the presence of the modification. For generation of the 3HA-OGT^{N567K} mESCs, we transfected the previously generated 3HA mESCs with pBABED puro U6 and pX335 (Cas9 D10A) vectors containing the gRNA sequences using the same procedure we previously used for the insertion of the 3HA-tag. Restriction enzymes and genomic DNA sequencing analysis were used for mutation identification. For the restriction fragment length polymorphism assay MouseGlasDiag_F and MouseGlasDiag_R primers were used to amplify by PCR the mutated site and the silent mutation which eliminates a *Hinf*I restriction site (**Supplementary Table S4**). The size of the bands following digestion (167 bp and 433 bp for wild type versus 600 bp for mutant) was used to screen for successful integration. Clones negative for cutting in the restriction enzyme assay were then sequenced to confirm the presence of the modification.

Immunocytochemistry

Cells were seeded in 0.1% gelatine coated coverslips and fixed with 4% (v/v) PFA in PBS. Fixed cells were permeabilized with PBS-T (PBS 0.5% Tween-20) twice and then blocked with PBS-T with 10% BSA [w/v]. Cells were further incubated with TUJ1 (anti-βIII-tubulin antibody, BioLegend) at 1:500, OGT antibody (ab96718, Abcam) at 1:500. Donkey anti mouse Alexa-488 and Donkey anti rabbit Alexa-568 (Thermo Fisher Scientific) were used as secondary antibodies at 1:500. DAPI reagent (Thermo Fisher Scientific) was used for nuclear staining at a final concentration of 1 μg/ml. Cells were mounted in Dako Fluorescence Mounting Medium (Agilent). Images were acquired on a Zeiss 710 confocal microscope and were processed using Image J (NIH) and OMERO.figure. Total neurite length per image was measured using the semi-automatic neuron tracing ImageJ plugin NeuriteTracer (6) and DAPI stained nuclei were counted in ImageJ using automated method. Average neurite length was calculated as the ratio between total neurite length and total nuclei number. Three biological replicates were performed and quantified. Student *t*-test were used to determine the significance of the difference.

Western Blot from mESCs

Cells were washed twice in PBS and harvested in RIPA buffer and the amount of protein was quantified using Pierce™ 660 nm Protein Assay Reagent (Thermo). 15-20 µg of cell lysate was loaded in NuPAGE 3-8% Tris-Acetate gels (Invitrogen) and transferred to Nitrocellulose membranes using wet transfer system. For neural differentiation, 15-20 µg of cell lysate was loaded on NuPAGE 4-12% Bis-Tris gels (Invitrogen) and transferred to PVDF-P membranes using wet transfer system. Membranes were blocked with TBS-T 5% BSA buffer [w/v]. Primary antibodies used were OGT (Sigma, F-12, 1:1000), OGA (Sigma, HP036141, 1:500), RL2 (Thermo, 1:1000), Oct3/4 (Santa Cruz Biotechnology, sc-5279, 1:500), Sox2 (Abcam, ab97959,1:1000), Tubulin (DSHB, 1:5000) and Histone 3 (CST, 1:1000)

Real Time PCR Analysis from mESCs

Total RNA extraction was performed with RNAeasy Kit (Quiagen) and then subjected to reverse transcription using 1000 ng of sample RNA and qScript cDNA Synthesis Kit (Quantabio) according to manufacturer's guidelines. qPCR was performed using Perfecta SYBR Green FastMix for iQ (Quantabio). All qPCR analyses were performed at 95 ºC for 30 seconds and then 40 cycles of 95 ºC for 5 s, 60 ºC for 15 s and 68 ºC for 10 s. The threshold crossing value was noted for each transcript and normalised to the internal controls GAPDH, Actb and Pgk1. The relative quantification of each mRNA was performed using comparative Ct method. Experiments were performed using CFX Connect Real-Time PCR Detection System (BioRad) and data processing was performed using CFX Manager Software (BioRad). Samples were assayed in triplicate using the thermocycler profile conditions described above. New dilutions for plasmid standard curves were prepared from stocks for each assay. Primers are listed in **Supplementary Table S1.**

Cloning of vectors coding for the guide RNA and repair template DNA for Drosophila CRISPR

The sxc^{N595K} *Drosophila* melanogaster line was generated using CRISPR as previously described (7) with some modifications. Briefly, a guide RNA site was selected with the help of the crispr.mit.edu online tool search and the annealing primer pair (gRNA_sxcT595_fwd and gRNA_sxcT595_rev) with appropriate overhangs for *Bpi*I restriction digestion were cloned into pCFD3-dU63gRNA

plasmid (8). A vector coding for repair template DNA of 2160 bp was generated from *Drosophila* Schneider 2 cell genomic DNA by PCR using GoTaq G2 Polymerase (Promega), T59fix_BAM_fwd and T59fix_NOT_rev primers (**Supplementary Table S2)**. The PCR product was inserted into pGEX6P1 plasmid. The desired mutation, in addition to four silent mutations (**Supplementary Figure S3**) was introduced by the BA_N595K_GLAS_F primer in a PCR reaction with BA_N595K_GLAShelp_R primer generating a 384 bp product. The silent mutations removed a *Hinf*I restriction site, thus enabling genotyping based on restriction digestion. The PCR product was subsequently utilized for restriction-free cloning (5) using KOD Hot start polymerase (Novagen). DNA products of cloning and mutagenesis were confirmed by sequencing.

Restriction fragment length polymorphism assay for genotyping sxc^{N595K} Drosophila line

To assess and confirm generation of CRISPR/Cas9 gene editing, candidate homo/heterozygous single embryos, wing of adult flies or whole flies were frozen and homogenized in 10-50 µl of DNA extraction buffer containing 10 mM Tris-HCl pH 8, 1 mM EDTA, 25 mM NaCl and 200 µg/ml freshly added Proteinase K (Roche) and subsequently incubated at 37 °C for 30 min, followed by inactivation of Proteinase K at 95 °C for 3 min, and centrifuged briefly. 1 µl of the crude DNA extract was used per 25 µl PCR reaction with T595_DIG primers (**Supplementary Table S2)**. 5 µl of PCR product was used for restriction fragment length polymorphism assay with *Hinf*I followed by agarose gel electrophoresis of the digested products. Samples that were resistant to *Hinf*I indicated CRISPR/Cas9 gene editing event and were sequenced using T595_DIG_SEQ primer.

Precise incorporation of the repair template into the right position of the genome was confirmed by sequencing a second round of PCR products obtained from potential homozygous CRISPR mutants with mixed T595_DIG and T595_oob primer pairs (**Supplementary Table S2)**.

Generation of the sxc^{N595K} lines, micro-injection and genetics

The *sxc^{N595K}* Drosophila lines were generated by CRISPR/Cas9 mutagenesis; a mixture of 100 ng/µl guideRNA plasmid with 300 ng/µl repair construct were injected into Vasa::Cas9 embryos in-house using an Eppendorf FemtoJet microinjector and Femtotip II. Founder male flies were crossed with

Sp/CyO balancer stock. Subsequently, single potential *sxc*^{N595K}*/CyO* germline mutant male flies and *Sp/CyO* virgins were crossed that allowed for elimination of the Vasa::Cas9 carrying X chromosome. Genotyping was carried out on F1 males. Stocks of *sxc*^{N595K}*/CyO* were established from F2 progeny. Furthermore, the genotype of *sxc*^{N595K} homozygotes derived from the *sxc*^{N595K}*/CyO* stock was confirmed by sequencing the corresponding genomic region. *sxc*^{N595K} lines were backcrossed to control genetic background for over 6 recombination steps. *w*^{1118} from Vienna Drosophila Resource Centre *(*VDRC #60000) was used as a control line.

We isolated two independent *sxc*^{N595K} knock-in lines (10.3 and 19.1). The *sxc*$^{N595K-19.1}$ line was fertile and viable as homozygote whereas the *sxc*$^{N595K-10.3}$ line was viable as homozygote but embryos from F1 generations did not develop to first larvae, as male *sxc*$^{N595K-10.3}$ flies were sterile. However, *sxc*$^{N595K-10.3}$*/sxc*^{1} and *sxc*$^{N595K-10.3}$/Df(2R)BSC630 male flies were fertile, indicating that the male sterile phenotype is independent of *sxc* gene and any alteration in the *sxc* gene region.

mRNA isolation, cDNA synthesis and sequencing of full-length Drosophila sxc cDNA

Drosophila 3rd instar larvae were dissected; imaginal discs and brain tissue were isolated into *RNAlater* RNA stabilization solution (Ambion). Samples were stored at 4 °C till RNA isolation. One volume PBS were added to aid sedimentation of tissue with centrifugation. RNeasy kit was used for isolating RNA including gDNA eliminator column (Qiagen). Approximately 40 μl RNA solution at 6-10 ng/μl concentration was obtained for each sample. For one step RT-PCR reaction 63.8 ng RNA was used in a 50 μl reaction volume using DmOGT_examine_F and DmOGT_examine_R primers in PrimeScript High Fidelity RT-PCR Kit (Takara). Cycling conditions were as follows – initial step of 50 °C for 30 min, 94 °C for 2 min. This was followed by 30 cycles of 98 °C for 10 sec, 55 °C for 5 sec and 72 °C for 3 min 30 sec. Samples of the reactions were visualized on a gel before the remainder of the reaction mixture was cleaned using the QIAGEN PCR clean-up kit. For sequencing approximately 500 base pair long fragments were amplified in PCR reaction using 'cDNA check' primers (**Supplementary Table S2**).

Western blotting from Drosophila samples

To prepare total lysates for Western blotting, embryos were collected on apple juice agar plates at 25 °C overnight (0-16 h). The embryos were dechorionated with bleach and snap frozen in liquid nitrogen. Fly heads from 3-5 days old 50-50% male-female flies were collected and separated from the fly body by vigorous vortexing for 2x 15 s. The frozen samples were homogenized in lysis buffer containing 1.5x NuPAGE LDS Sample Buffer (Invitrogen), 50 mM Tris- HCl (pH 8.0), 150 mM NaCl, 4 mM sodium pyrophosphate, 1 % TritonX 100, 1 mM EDTA, 1 mM benzamidine, 0.2 mM PMSF, 5 µM leupeptin and 1% 2-mercaptoethanol. Lysates were then heated for 5 min at 95 ºC, centrifuged at 13000 rpm for 10 min and supernatants were collected. Protein concentrations were estimated using the Pierce 660 nm protein assay supplemented with Ionic Detergent Compatibility Reagent (Thermo Scientific). Protein concentrations were adjusted across samples. 25 µg of protein lysate was loaded on NuPAGE 4-12% Bis-Tris protein gels (Invitrogen) and transferred onto nitrocellulose membrane. Membranes were developed with mouse anti-O-GlcNAc antibody, RL2 (1:1000, Thermo), rabbit anti-OGT (1:1000, Abcam, ab-96718) and rabbit anti-actin (1:5000, Sigma, A2066) primary antibodies and donkey anti-mouse IgG 800 and goat anti-rabbit IgG 680 infrared dye conjugated secondary antibodies (Li-Cor, 1: 10000).

References

1. Pathak S, et al. (2015) The active site of O-GlcNAc transferase imposes constraints on substrate sequence. *Nat Struct Mol Biol* 22(9):744–749.
2. Kabsch W (2010) XDS. *Acta Crystallogr D Biol Crystallogr* 66(Pt 2):125–32.
3. Winn MD, et al. (2011) Overview of the CCP4 suite and current developments. *Acta Crystallogr Sect D Biol Crystallogr* 67(4):235–242.
4. Hodgkins A, et al. (2015) WGE: a CRISPR database for genome engineering. *Bioinformatics* 31(18):3078–80.
5. van den Ent F, Löwe J (2006) RF cloning: A restriction-free method for inserting target genes into plasmids. *J Biochem Biophys Methods* 67(1):67–74.
6. Longair MH, Baker DA, Armstrong JD (2011) Simple Neurite Tracer: open source software for reconstruction, visualization and analysis of neuronal processes. *Bioinformatics* 27(17):2453–2454.
7. Mariappa D, Ferenbach AT, Daan MFVA (2018) Effects of hypo-o-glcnacylation on drosophila development. *J Biol Chem* 293(19):7209–7221.
8. Port F, Chen H-M, Lee T, Bullock SL (2014) Optimized CRISPR/Cas tools for efficient germline and somatic genome engineering in Drosophila. *Proc Natl Acad Sci* 111(29):E2967–E2976.
